# Use of mHealth Technology for Patient-Reported Outcomes in Community-Dwelling Adults with Acquired Brain Injuries: A Scoping Review

**DOI:** 10.3390/ijerph18042173

**Published:** 2021-02-23

**Authors:** Shannon B. Juengst, Lauren Terhorst, Andrew Nabasny, Tracey Wallace, Jennifer A. Weaver, Candice L. Osborne, Suzanne Perea Burns, Brittany Wright, Pey-Shan Wen, Chung-Lin Novelle Kew, John Morris

**Affiliations:** 1 UT Southwestern Medical Center, Department of Physical Medicine & Rehabilitation, Dallas, TX 75390, USA; andrew.nabasny@utsouthwestern.edu (A.N.); candice.osborne@utsouthwestern.edu (C.L.O.); brittany.wright@utsouthwestern.edu (B.W.); novelle.kew@utsouthwestern.edu (C.-L.N.K.); 2 UT Southwestern Medical Center, Department of Applied Clinical Research, Dallas, TX 75390, USA; 3 Department of Occupational Therapy, University of Pittsburgh, Pittsburgh, PA 15219, USA; lat15@pitt.edu; 4 Shepherd Center, Atlanta, GA 30309, USA; Tracey.Wallace@shepherd.org (T.W.); John.Morris@shepherd.org (J.M.); 5 Department of Clinical Research & Leadership, George Washington University, Washington, DC 20006, USA; jenweaver524@gwu.edu; 6 School of Occupational Therapy, Texas Woman’s University, Denton, TX 76204, USA; sburns3@twu.edu; 7 Department of Occupational Therapy, Georgia State University, Atlanta, GA 30303, USA; pwen@gsu.edu

**Keywords:** mHealth, rehabilitation, brain injury, measurement, patient-reported outcomes

## Abstract

The purpose of our scoping review was to describe the current use of mHealth technology for long-term assessment of patient-reported outcomes in community-dwelling individuals with acquired brain injury (ABI). Following PRISMA guidelines, we conducted a scoping review of literature meeting these criteria: (1) civilians or military veterans, all ages; (2) self-reported or caregiver-reported outcomes assessed via mobile device in the community (not exclusively clinic/hospital); (3) published in English; (4) published in 2015–2019. We searched Ovid MEDLINE(R) < 1946 to 16 August 2019, MEDLINE InProcess, EPub, Embase, and PsycINFO databases for articles. Thirteen manuscripts representing 12 distinct studies were organized by type of ABI [traumatic brain injury (TBI) and stroke] to extract outcomes, mHealth technology used, design, and inclusion of ecological momentary assessment (EMA). Outcomes included post-concussive, depressive, and affective symptoms, fatigue, daily activities, stroke risk factors, and cognitive exertion. Overall, collecting patient-reported outcomes via mHealth was feasible and acceptable in the chronic ABI population. Studies consistently showed advantage for using EMA despite variability in EMA timing/schedules. To ensure best clinical measurement, research on post-ABI outcomes should consider EMA designs (versus single time-point assessments) that provide the best timing schedules for their respective aims and outcomes and that leverage mHealth for data collection.

## 1. Introduction

In the United States, approximately 3.5 million people experience a traumatic brain injury (TBI) [1] and 795,000 experience a stroke each year [2]. Acquired brain injury (ABI) including traumatic and non-traumatic brain injury (e.g., stroke) often results in secondary health issues [3,4,5], some of which may persist for years after the initial injury event [6,7]. Individuals with ABI often experience multiple barriers to receiving care and managing long-term symptoms, including inconsistent long-term follow-up with providers, limited access to community-based healthcare services [8,9], and costs associated with rehabilitative care and transportation [10]. 

Barriers to long-term clinical surveillance also hinder care. These challenges include reliance on self-report, length and breadth of available validated measures, complexity of administration, and ecological validity. Symptom-reporting also frequently relies on retrospective reporting, requiring individuals with ABI to think back over the past several months or longer, which make them prone to reporting errors as a result of impaired self-awareness or recall bias commonly associated with TBI-related cognitive impairment. However, self-report is still the most efficient and practical method for community-based clinical surveillance of patient-reported outcomes. Increasing measurement frequency and focusing on individuals’ immediate experiences, as done through Ecological Momentary Assessment (EMA) [11], may also reduce these limitations of self-reporting. 

Given the ubiquity of smartphone use and the ability to access this technology in one’s community, mobile technology may have the potential to be a cost-effective way to improve and support symptom surveillance, access to care, self-management, and long-term outcomes. Most Americans, including individuals with brain injury [12], own smartphones [13]. Further, research suggests that mHealth technology, readily available in most people’s pockets, may effectively promote behavior change [14,15].

Through mobile health (mHealth) technology, healthcare providers can prospectively collect and/or share health-related information (e.g., tracking symptoms, documenting health-related activities/events), improving communication between patients and healthcare providers. If communication via mHealth occurs in only one direction (clinicians sending information out via a device *or* individuals with ABI inputting information into an app or device), this is considered a one-way system. A two-way systems allows for back and forth exchange of information and is preferred by ABI survivors, their family members, and clinicians [16]. 

To date, though, there is limited evidence to support the efficacy of mHealth technologies to improve functional outcomes following an ABI [17,18,19,20]. While thousands of mHealth applications are currently available for download, fewer than 1% of these are grounded in evidence-based knowledge [15,21]. Furthermore, only a minority of persons with brain injury report a clinician having discussed mHealth services with them following their injury [22,23]. And a recent survey of over 500 rehabilitation clinicians revealed only 23% felt knowledgeable about rehabilitation technology currently available and only 51% reported being comfortable using such technology in their practice [24]. But when presented with mHealth applications, most TBI and stroke survivors say that they would use these technologies and believe these applications would help improve their symptoms [16,25,26]. This demonstrates a critical gap in rehabilitative care: when individuals are willing to use mHealth technologies, but do not receive informed recommendations from trusted, knowledgeable healthcare providers, there is a greater risk that they may use applications inappropriate for their rehabilitation goals.

Studies using personal digital assistants (PDAs) and telephone-based interventions suggest that mHealth interventions are both feasible and potentially efficacious for managing chronic symptoms in individuals with brain injuries [23,27]. Two recent reviews on mHealth use after ABI found that while there is insufficient evidence to support the use of mHealth interventions as standard practice, emerging evidence does support their efficacy for improving goal attainment and social participation [20,28]. Both reviews highlight the importance of developing more personalized, patient-centered mHealth interventions sensitive to the needs and abilities of individuals with ABI. Evidence further supports the use of EMA for measuring psychological health outcomes [29]. The repeated sampling used in EMA captures patterns in an individual’s mood, activities, symptoms, and behaviors in real-time and in an individual’s natural environment [11]. EMA via mHealth could reveal moments for in situ intervention (Ecological Momentary Intervention [EMI]) [30], promoting stable long-term changes in behavior when combined with mHealth interventions.

MHealth technology may be promising for alleviating symptoms and improving long-term outcomes among individuals with chronic conditions. However, there remains insufficient evidence for definitive recommendation of their use after ABI. A previous review presented promising evidence for mHealth interventions in community-dwelling individuals with TBI [20], but no such review examines the evidence for mHealth technology to support collecting patient-reported outcomes after ABI. Therefore, the purpose of this review is to examine the currently available literature and evidence on the use of mHealth technology for long-term assessment of patient-reported outcomes in community-dwelling individuals with chronic ABI. We discuss the state of the science and limitations of these technologies and lay out recommendations for future research in this area. 

## 2. Materials and Methods

We conducted a scoping review of the literature, following the Preferred Reporting Items for Systematic reviews and Meta-Analyses extension for Scoping Reviews (PRSIMA-ScR) [31] guidelines and 5-stage framework described by Arksey and O’Malley [32]. Stage 1, defining the research question and scope, begun at a meeting of authors during the American Congress of Rehabilitation Medicine Annual Meeting in 2018 and completed in the Summer of 2019. 

Stage 2 included determining eligibility criteria and identifying relevant studies. Eligibility criteria for articles to be included in this review were: (1) Sample population was primarily acquired brain injury (traumatic brain injury, concussion, stroke, other brain injury); (2) Sample population included all ages and both civilians and veterans; (3) Assessment was collected via mobile phone/device (e.g., not just internet/computer-based), though could be done via app or text messaging; (4) Self-reported or caregiver-reported assessments of patient-reported outcomes (e.g., not direct collection of physiologic measures, activity data, etc.); (5) Assessment occurred in the community (e.g., not exclusively in a clinical setting); (6) Published in English; and (7) Published in or after 2015 (search conducted in August 2019 with the goal of examining research in the past 5 years). Systematic reviews, study protocol papers, and conference abstracts were excluded. 

Stage 3 was identifying relevant studies through a database search. We worked with a research librarian to conduct a search on 16 August 2019 of the following databases: Ovid MEDLINE(R) < 1946 to 16 August 2019, MEDLINE InProcess, EPub, Embase, & PsycINFO. The exact search terms are presented in Supplemental Table S1. Our search strategy was limited to the English language, research involving humans, and publications from January 2015 to 15 August 2019. 

Two reviewers independently examined all titles and abstracts for full text review, with a third reviewer making determinations when there were questions regarding inclusion or when the two reviewers did not have consensus. Two different reviewers completed full text review of identified articles for final inclusion. Another study author engaged in hand-searching reference lists of included articles and studies reviewed in previous systematic or scoping reviews for additional articles not identified in the database search.

Stage 4, data extraction, was completed by four authors who confirmed eligibility and completed data extraction for included articles (following pre-identified data elements in Table 1 and Table 2). Stage 5, summarizing and reporting results followed.

## 3. Results

**Figure 1:**
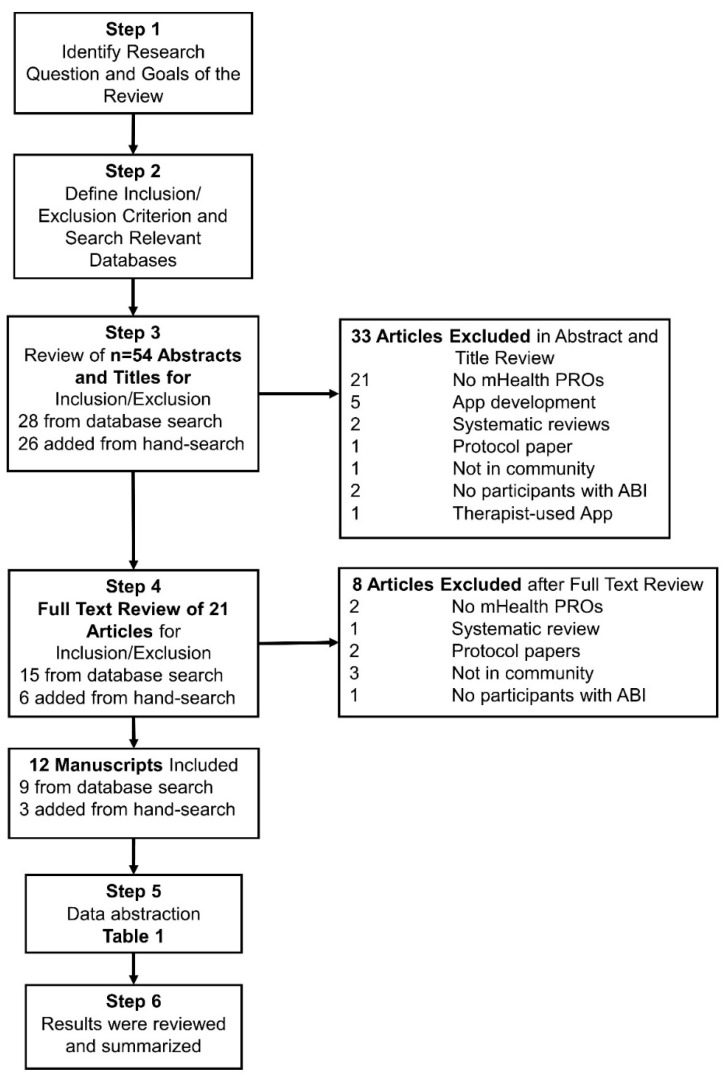
Flow chart of study inclusion and exclusion.

Figure 1 outlines 5-stages of this review, including a summary of the number of articles identified from the initial database search and hand-searching (*n* = 54, Step 3) through the final number of articles included in the review (*n* = 12) [33,34,35,36,37,38,39,40,41,42,43,44]. 

### 3.1. Characteristics of Included Studies

Table 1 presents details of the sample, constructs measured, and study design. Table 2 presents details of the mHealth technology used, inclusion of EMA, purpose of the study, and results for the 12 included manuscripts (representing 11 distinct studies), by TBI (grey shaded rows) and stroke (white rows). Five manuscripts included only individuals with stroke; six manuscripts included only individuals with TBI, though two were from the same parent study; and one manuscript included individuals with stroke and TBI. Of the five unique studies focusing on TBI, three included adolescents with concussion [35,36,37], one included service members (injury severity unknown) [33], and the other (from which two manuscripts were derived) included adults with complicated mild to severe TBI [34,38]. The study including adult participants with either TBI or stroke did not specify injury severity [40]. Of the five studies focusing on stroke [39,41,42,43,44], two also included caregivers [41,44]. A total of 219 people with TBI and 171 people with stroke (plus 43 of their caregivers) participated in the 12 studies.

### 3.2. Summary of mHealth Technology Used and Constructs Measured

Ten studies used smartphone apps: five were one-way apps and five were two-way communication apps. Of the remaining two studies, one used text messaging linked to a web-based clinician management system and the other used a personal digital assistant. Constructs measured via mHealth included affect, anxiety, and/or depressive symptoms (five studies), daily and social activities (five studies), fatigue (three studies) post-concussive symptoms (three studies), disability (one study), stroke risk factors (one study), and cognitive exertion (one study). 

### 3.3. Inclusion of Ecological Momentary Assessment via mHealth

Ten studies (11 papers: six in TBI, four in stroke, one in both) included EMA, with frequency and duration of EMA varying considerably. Four studies examined once a day measures (some at fixed times, some at random times), with a fifth study examining event-related reporting with a target of once a day for five of seven days each week. The remaining five studies examined multiple times per day measures (3, 5, and 10), some at fixed times and some at random times. Duration of EMA ranged from 6 days to 6 months: three studies included 6–7 days of multiple assessments per day, one study included multiple assessments per day for up to 18 days, one study included multiple assessments per day for two weeks, one study included daily measures for 3 weeks, two studies included daily measures for 8 weeks, one study included daily measures for 36 weeks, and one study included daily assessments for 6 months. Two studies examined the psychometric properties of the previously validated assessments when collected via EMA [34,41].

### 3.4. Traumatic Brain Injury Studies and Findings

Patient-reported outcomes after TBI measured mood, affect, anxiety, pain, fatigue, post-concussion symptoms (including post-concussive headache), activity, recovery, and overall well-being. Only one study compared patient-reported outcomes via the app (daily activity) to a biometric measure (data collected via accelerometer) [35].

For TBI, reported compliance with assessments ranged from 50–82% overall [33,34,35,37]. Pavliscsak et al. noted that those with behavioral problems, younger participants, and those with lower well-being were less compliant (many <50%) and took longer to respond to EMA prompts than those without behavioral problems, older participants, and those with higher well-being, regardless of presence or absence of TBI or PTSD [33]. Juengst et al. though finding an overall high average compliance for daily assessments over 8 weeks (73.4%), noted a bimodal distribution in compliance, suggesting that participants generally either did or did not complete daily reports rather than a middling compliance across all participants [34]. Finally, Worthen-Chaudhari et al., reported that using a gamified app may facilitate better compliance, particularly among adolescents [36]. 

Juengst et al. noted significant within person variability in daily mood, affect, and fatigue reporting over 6–8 weeks, despite no intervention, supporting the need for EMA—rather than a single time point—to capture these patient-reported outcomes [38]. Variability from day to day was greater for women and those reporting more symptoms. Sufrinko et al. also noted that participants reported fewer symptoms in the morning than the afternoon or evening [37], supporting EMA multiple times per day or at variable times from day to day to capture natural diurnal variation. They also found that symptom scores derived from EMA were better at predicting time to recovery after concussion than symptom scores obtained at clinic visits [37]. Worthen-Chaudhari et al. examined the effects of concussion symptom tracking as intervention, finding that those who more consistently reported their symptoms through the gamified app also noted more symptom improvement over time [36]. Wiebe et al. examined time-lagged relationships between concussion symptoms and both cognitive exertion and physical activity in youth concussion, finding that more cognitive exertion was associated with more concussion symptoms on the same day and for 2 days following and that, conversely, more physical activity was associated with fewer concussion symptoms on the same day and the 2 days following [35]. This is contrary to what Lenaert et al. reported in their study including both adults with TBI and stroke wherein they found that physical activity and fatigue predicted more negative affect on the same day; however, it did not predict negative affect on subsequent days [45]. 

### 3.5. Stroke Studies and Findings 

Patient-reported outcomes after stroke measured daily and social activities, disability, stroke risk factors, depression, anxiety, and fatigue. Only one study directly compared agreement between a measure (the modified Rankin Scale) assessed in the clinic to the same measure assessed via mHealth, noting 85% agreement if scores were dichotomized to reflect functionally independent versus dependent [41]. 

Similar to results from studies in TBI, three studies using mHealth for EMA demonstrated the importance and utility of this approach over single time point measures. Seo et al. found that daily reporting of stroke risk factors led to an overall shift in risk profiles [42], suggesting that daily tracking led to behavior change. Villain et al. used EMA measures to examine change in depression symptoms and activities of daily living participation over time, allowing them to examine early predictors of positive change over time. They found that more material support (e.g., provision of items or task assistance) and higher quality of moral support during the hospitalization led to later improving activities of daily living participation and decreasing depressive symptoms [43]. Lenaert et al. conclude that Experience Sampling Methods (conceptually equivalent to EMA) are needed to design personalized rehabilitation programs after stroke, after finding that more effortful activities or physical activities early in the day were associated with later fatigue, but that fatigue was highest when participants were resting or doing nothing [39]. Understanding differences between concurrent and time-lagged associations could inform use of energy conservation interventions and timing of EMI (in the moment interventions) to prevent and reduce fatigue post-stroke.

For stroke, reported compliance with assessments ranged from 50–77.3% overall. The consensus across studies was that mHealth was feasible and acceptable, with potential to improve symptoms and health. Fors et al. reported overall positive opinions about use of mHealth to track daily activities, from both patients with stroke and their family members [44]. In the absence of tech support, older participants struggled more with technical issues, with many relying on family members to send and receive text messages [44].

## 4. Discussion

The purpose of the current review was to examine recent (2015–2019) literature to describe use of mHealth technology, EMA design considerations, and findings in studies of patient-reported outcomes in community-dwelling individuals with chronic ABI. We found 12 manuscripts representing 11 distinct studies that used mHealth technology for community-based clinical symptom surveillance of affect, anxiety, and/or depression, daily and social activities, fatigue, post-concussive symptoms, disability, stroke risk factors, and cognitive exertion. There was variability across studies with respect to the constructs measured and frequency, duration, and timing schedule of EMA data collection. As a result, we cannot make recommendations as to the optimal content, timing, or mHealth platform for capturing accurate information and avoiding participant fatigue. However, personalization is a consistent theme across studies in this review and past studies on mHealth use after ABI [20,28], and individual differences will likely require personalized approaches that pair mHealth technology with unique needs and abilities of each person.

Despite differences across studies, we found consistency in advantages of using EMA that are not present when collecting single time-point measures, especially for those symptoms for which fluctuating frequently over short periods of time is common. To analyze EMA data meaningfully, it is important to organize the data using a timing schedule that aligns with the research question [46]. Our review uncovered a variety of EMA designs, including event-contingent assessments and random or fixed assessments either once or multiple times throughout the day. Another design that may be helpful to researchers is a combination of these approaches, such as event-related reporting plus either a fixed or random timing schedule as a complimentary way to collect data. For example, a researcher may ask the participant to report when feeling a panic attack (event-related reporting), but then also collect data throughout the day at fixed or random times to uncover the participant’s affect/mood leading up to or directly after that event. We also noticed that the number of days for data collection varied considerably amongst the EMA studies in our review. This can be especially important for research questions that are interested in capturing changes in effects over time. Researchers wishing to examine temporal changes in outcomes should consider the implications of nature within-person variability over the period of data collection that may mask or be misinterpreted as meaningful change if single time point measures are used. For example, we may observe day to day variability in mood across the week, so week-long daily data capture may provide more insight for pattern recognition over time. Future researchers should consider the aims and outcomes of the research study, the characteristics of the population, and the strengths and limitations of existing EMA literature when selecting an EMA design.

While papers included in our review clearly provide support for using mHealth for EMA at multiple, varied times per day, scarce detail is offered on sampling in varied environmental contexts. Assessment in natural environments is a core benefit of implementation of EMA in the community, and assessment of contextual factors influencing health, function, and participation is a key construct in rehabilitative care [47]. When EMA is used to assess aspects of psychological and behavioral function, implementation in the community may support identification of antecedent and proximal events influencing behavior in real world situations, thereby supporting self-awareness and delivery of timed-right, personalized interventions based on assessment [11,48,49]. However, some community settings and activities may present barriers to EMA. For example, an adolescent may not be able to receive or respond to notifications to complete an assessment when they are in school or adults may not be able to respond while at work. Likewise, the same may apply to an adult who is driving or engaged in childcare tasks. And regardless of setting, those without consistent access to high-speed connectivity may not have the network connectivity needed to support technical aspects of data collection and data transfer for some mHealth assessments [50]. Finally, implementing EMA in the community may raise concerns regarding remote monitoring of safety events in cases where collected data are not remotely monitored in real time but are instead stored and later viewed by healthcare providers during visits. And some patients may have added privacy concerns in cases in which sensitive health data is collected or location tracking is used [51]. 

In general, patient-oriented mHealth apps are designed to assist patients in managing their own health or to support the provision of healthcare services when a face-to-face encounter is not feasible [52,53,54,55]. Hundreds of thousands of mHealth apps exist, and many are effective in maintaining or improving health outcomes [56,57]. However, the majority of these apps are not designed for individuals with disabilities, and an even smaller number have been evaluated for accessibility by patients who experience upper extremity limitations, visual or cognitive deficits, aphasia, or emotional or behavioral disturbance, all common after ABI [58]. Our findings suggest that few mHealth apps have been designed specifically for collecting patient-reported outcomes in the ABI population, yet those that have demonstrate initial acceptability and potential efficacy. It is likely that evidence-based mHealth apps will be integrated in rehabilitation clinical practice in the near future, though currently it is challenging for rehabilitation practitioners and patients to identify evidence-based apps that are accessible and effective [59]. A 2018 systematic review of studies that applied mHealth evaluation methods or an assessment approach for mHealth apps revealed seven criteria for clinicians to use in assessing the appropriateness of mHealth apps: (1) design, (2) information/content, (3) usability, (4) functionality, (5) ethical issues, (6) security and privacy and (7) user-perceived value [60]. It is also paramount that rehabilitation practitioners include the individual patient in the decision to implement app use into the patient care plan [59], and that there be a practitioner following individuals with ABI who are using mHealth apps after discharge from clinical settings [16]. 

Healthcare providers may be afforded the opportunity to prospectively collect client information and share one-direction or bi-directional information with clients. Providers may use mHealth technology with an overarching aim of improving ABI recovery outcomes among their clients living in the community. While various mHealth technologies have been developed and tested, our study reveals that almost no articles have focused on use of this technology across the transition from acute or sub-acute care to the community. Although self-management largely occurs during community living, implementing mHealth technology across the care continuum has the capacity to improve adoption and use. However, only two papers in our review included mHealth technology started during the acute or sub-acute care period, and in one of these, only training was completed before transition to the community. Four articles excluded from our review for occurring exclusively in clinical settings, conversely, did not continue out into the community. Users may be described on a continuum of downloading and never using mHealth technology, discontinuing it immediately after use, or even using the technology in unexpected ways [61]. Careful consideration should be given to providing appropriate training to support use; our stroke findings suggest this may be especially true in older adults. Practitioners may be able to support use and adoption with strategies which include: (1) supporting the identification of evidence-based apps for self-assessment, (2) matching the technology to the specific user wants and needs, and (3) providing hands-on opportunities for practice and training with the technology itself. Furthermore, providers may be able to guide clients in accessibility interfaces and add-ons that may be needed considering the vast range of impairments (e.g., visual, motor, cognitive) people may experience after ABI [62]. These integration and training elements may be frequently over-looked but could be easily integrated into existing treatment plans based on client-centered needs across the care continuum. Introducing mHealth technology prior to discharge or with a provider familiar with unique needs of each client may be useful in supporting adoption and long-term use of the technology to support improved outcomes. 

### Limitations

Despite integrating a comprehensive search strategy, a major limitation of this study is that we have missed published articles that met the pre-defined inclusion criteria. This limitation may be associated with database selection, search terms used, and the search timeline. Additionally, we did not include articles written in languages other than English and this may have limited the findings of this study. We also did not include conference abstracts due to their limited information for data extraction. Although we intended to include studies that included individuals with all types of ABI, we did not find any studies that enrolled individuals with brain injuries other than TBI or stroke. Therefore, our findings might not be generalizable to people with brain injuries other than TBI and stroke.

## 5. Future Directions

Going forward, research on community-based EMA using information and communication technology, should focus on: (1) strategies for engaging survivors and their caregivers; (2) processes for adapting EMA to individual progress or decline; (3) clinician needs and acceptance (including impact on clinician workflow); (4) emerging capabilities in cloud-based artificial intelligence (AI), chat and natural language processing (NLP). 

A key challenge to mHealth, particularly for self-assessment and self-management, is keeping the survivor (and caregiver) sufficiently engaged in processes that are often repetitive, like answering questions about their status or completing home exercise programs. At minimum, individuals need feedback on how they are doing and knowledge that someone is monitoring their performance, which reinforces accountability [63]. Beyond this minimum, other techniques for patient engagement could include the use of challenges and rewards to complete self-assessments. Such “gamification” in healthcare is more typically associated with interventions like exercise [64], smoking cessation [65], and weight loss [66] than self-assessments and may be more difficult to implement for people with brain injury. However, Worthen-Chaudhari et al.’s study, included in this review, demonstrates that gamification is potentially feasible in terms of compliance and effective for improving symptom management, at least for youth concussion [36]. They framed factors tracked by the app as “bad guys”, “power ups”, or “allies”; “bad guys” were factors that may trigger symptoms or maladaptive responses, “power ups” were effective strategies, and “allies” were members of a user’s social network who could send encouragement. Not only did they employ feedback and tracking for how often user’s conquered or did not conquer a “bad guy” each day, but they also employed social networking to improve compliance and symptom-management [36]. Still, research on engagement strategies, including challenges and virtual rewards for compliance, could yield insights into methods for increasing compliance and the types of patients with brain injury who may be most responsive. Updating the content, timing, and frequency of the assessments based on individual progress or decline is also critical and related in part to the challenge of engagement in ongoing self-assessments. A static EMA script or form, and the timing and frequency of assessments, may grow less useful for brain injury survivors as they improve. Additionally, reporting needs may differ based on available resources, including both internal and external resources. Research is needed on EMA programs that can adapt to patient progress (or regression) and reporting needs.

For clinicians, more research is needed on how they want to access data, how the data are presented, and impact on workflow. Research in various countries show that while clinicians embrace the potential of health technologies (mobile and otherwise), they remain especially concerned about how it may affect workflow [16,67,68,69]. When should they review patient data? How frequently? Will they be reimbursed for the time they spend reviewing remotely collected patient self-assessment data? In the United States, 2020 updates to mHealth and patient monitoring reimbursement policies by the Centers for Medicare and Medicaid Services (CMS, the largest government healthcare insurer in the U.S.) do not include coverage for remote patient self-assessment [70]. More evidence of effectiveness is needed for EMA to be reimbursed by public and private insurers in the U.S. and other countries.

Finally, rapid advances in cloud computing, AI, chat, predictive analytics and NLP offer new opportunities for EMA in the home and community. Gartner predicts that by 2021, customers will manage more than 85% their relationship with a business without interacting with a human, and that 15% of all customer service interactions will be completely handled by AI [71]. Furthermore, “[i]n the “post-app era,” chatbots will become the face of AI and bots will transform the way apps are built,” according to Gartner [72]. While AI, chat and NLP offer new capabilities, they also create complexity for EMA development and testing. Additionally, cloud-based AI solutions may not be feasible for low-resource patients or for localities lacking access to high-speed internet. Still, these technologies offer new capabilities for EMA, allowing assessments to be more detailed, adaptive (during an individual self-assessment and over a series of assessments), engaging and accessible by adding voice interaction to visual and tactile interfaces. 

## 6. Conclusions

Consistent with similar findings of preliminary evidence on the use of mHealth technology for intervention after TBI [20], this scoping review showed promising early evidence for the feasibility of using mHealth technology for patient-reported outcomes in community-dwelling adults with chronic ABI. The advantage of mHealth technology for community-based assessment is its ability to easily and efficiently conduct EMA—and eventually, EMI—in individuals’ natural environments, while still maintaining contact with and oversight from a trained clinician. ABI survivors and their caregivers have noted the importance of and their preference for a “human component” of mHealth [16]—that is, they want a person to actually receive the responses they punch into an app and they want to get responses in return. Technology continues to develop faster than the research on its feasibility, usability, and efficacy, but the importance of clinicians having available evidence to support clinical recommendations for use of mHealth to support ABI survivors cannot be understated.

## Figures and Tables

**Table 1:** Study characteristics.

Authors (Year) [Reference]	Title	Sample	Construct/s & Measures	Design
Traumatic Brain Injury				
**Pavliscsk et al. (2016)** [33]	Assessment of patient engagement with a mobile application among service members in transition.	***n*** = 95 service members **Gender:** 80 male; 15 female **Average Age:** 34.7–39.7 **Severity:** unknown **Chronicity:** unknown **Other:** participants grouped by health status (see below) ***n*** = 40 w/o Behavioral Health Problem **Gender:** 33 male;7 female **Average Age:** 39.7 (10.4) ***n*** = 22 w/ Behavioral Health Problem(s) Only **Gender:** 16 male; 6 female **Average Age:** 34.7 (10.3) ***n*** = 24 w Behavioral Health Problem(s) Plus PTSD and/or TBI **Gender:** 22 male;2 female **Average Age:** 37.7 (10.2) ***n*** = 9 w/ PTSD and/or TBI **Gender:** 9 male; 0 female **Average Age:** 38.7 (12.3)	**Participant engagement** Percentage of questionnaires responded to each week. Number of hours between when a questionnaire synced with a participant’s phone and when they responded.	Secondary data analysis of one arm of a multisite prospective RCT. Frequency of Assessment: Participants received 7 daily questionnaires (each sent once per week) for up to 36 weeks.
**Juengst et al. (2015)**	Pilot feasibility of an mHealth system for conducting ecological momentary assessment of mood-related symptoms following traumatic brain injury.	***n*** = 20 community-dwelling adults with TBI (3 completed baseline assessments only). **Gender:** 12 male; 8 female **Average Age:** 36.7 (12.4) **Average Time Since Injury:** 5.2 (3.6)	**Feasibility:** Compliance: days completed divided by days scheduled. Satisfaction: 6 questions on 7-point Likert scale. Usability: Telehealth Usability Questionnaire (TUQ), assessed via phone at last call. **Mood-related:** PHQ-2: Daily mood GAD2: Daily mood PANAS: Daily & biweekly affect PHQ-9: Baseline & biweekly mood. GAD-7: Baseline & biweekly anxiety.	Prospective, repeated-measures. Mood-related symptoms assessed at Baseline, and over 8 weeks using the iPerform app and biweekly phone interviews.
**Wiebe et al. (2016)**	Ecologic Momentary Assessment to Accomplish Real-Time Capture of Symptom Progression and the Physical and Cognitive Activities of Patients Daily Following Concussion	***n*** = 34 youth with concussion. **Gender:** 18 male; 16 female **Median Age:** 15 (IQR 13–16) **Median Time Since Injury:** 6 days (IQR 3–10)	**Concussion Symptoms** Post-Concussion Symptom Scale **Daily Activities** Activity questionnaire **Cognitive Exertion** Number of texts sent Minutes of screen time & gaming Minutes of reading or schoolwork	Baseline in person and then two weeks of daily (several times per day) symptom and activity reporting
**Worthen-Chaudhari et al. (2017)**	Reducing concussion symptoms among teenage youth: phase I and II evaluation of a mobile health app	**Phase I:** ***n*** = 20 youth with concussion **Average Age:** 16.6 +/− 1.6 years **Gender:** Males 6 (30%) **Phase II:** ***n*** = 22 youth with concussion **Average Age:** 15.6+/− 1.7 years **Gender:** Males 5 (22.7%)	**Concussion Symptoms** SCAT-3: Post-concussion symptoms CES-DC: Depression Life Orientation Test-Revised: Optimism	Phase I and Phase II open-label, non-randomized trial Event-related reporting with a target of 1x daily for 5/7 days per week for 3 weeks.
**Sufrinko et al. (2019)**	Mobile Ecological Momentary Assessment of Post-concussion Symptoms and Recovery Outcomes.	***n*** = 20 athletes with sports-related concussion (SRC) **Sex:** 12 male; 8 female **Age Range:** 12–19	**Post-Concussion Symptoms** Post-Concussion Symptom Scale Neurocognitive Testing Vestibular/oculomotor screening (VOMS) **“Activity Type”:** cognitive, physical, sedentary, or vestibular. **“mEMA symptom score”** (modified PCSS, excluding nighttime sleep symptoms): total symptom score, symptom factor scores (cognitive-migraine-fatigue, somatic, affective). **“Patient recovery duration”:** days until medical clearance.	Prospective study. Visit 1—<72 h post-injury. Included clinical testing measures. Visit 2—6–18 days post-injury. Included clinical testing measures. mEMA questionnaire—surveys sent at 3 fixed times blocks (morning, afternoon, and evening), every day until medical clearance or second follow-up (whichever came first). Included activity type and mEMA symptom score measures.
**Juengst et al. (2019)**	Variability in daily self-reported emotional symptoms and fatigue measured over eight weeks in community dwelling individuals with traumatic brain injury.	***n*** = 18 people with TBI **Average Age** =38.3 (22–60), **Gender:** 10 men; 8 women	**EMA of mood:** Patient Health Questionnaire-2 Generalized Anxiety Disorder-2 Positive and Negative Affect Schedule (10 items) Fatigue Impact (1 item)	Secondary data analysis Participants were in a feasibility study using smartphone to assess daily mood-related symptoms in m-s TBI. Mood related symptoms were collected for 8 weeks daily.
**TBI and Stroke**				
**Lenaert et al. (2018)**	Exploring the feasibility and usability of the experience sampling method to examine the daily lives of patients with acquired brain injury	**In the Netherlands:** ***n*** = 17 adults with stroke or traumatic brain injury **Stroke:** *n* = 9 **TBI:** *n* = 8 **Age range:** 18–65 Average Age: 44.2 (14.5) **Gender:** Males 8 (47%)	**Mood:** Positive & Negative Affect Schedule (PANAS) **Location** **Social Context** **Activities** **Physical well-being** (including fatigue)	Longitudinal observational study. 10 random moments daily for 6 days to measure mood, location & context, activities, and well-being.
**Stroke**				
**Cooray et al. (2015)**	Mobile Phone-Based Questionnaire for Assessing 3 Months Modified Rankin Score After Acute Stroke: A Pilot Study.	***n*** = 48 acute stroke patients and/or their caregivers **Responses from:** Patients only: 16 Patients and Caregivers: 6 Caregivers only: 26 **Gender/Sex (unsure which):** 30 male; 18 female **Average Age:** 67	**Modified Rankin Scale (mRS):** measure of functional level (degree of disability/dependence) after stroke. **Rankin Focused Assessment (RFA):** structured form of the mRS (reduces inter-rater variability). Translated to Swedish.	Cohort study Participants were reminded via monthly messages in the app of participation. At 3 months, participants were sent RFA via the app. Within 1 week after RFA, personnel blinded to participants’ scores rated participants using mRS in clinic.
**Seo et al. (2015)**	Feasibility of using a mobile application for the monitoring & management of stroke-associated risk factors.	***n*** = 48 **Average Age:** 52.65+/− 10.25 years **Gender:** Males 36 (75%)	**Daily reports and measures of risk factors for stroke:** Blood pressure Medication adherence HbA1c Waist circumference Number of cigarettes smoked	Prospective, single-center, single-arm, open-label clinical trial
**Villain et al. (2017)**	Very early social support following mild stroke is associated with emotional and behavioral outcomes three months later.	***n*** = 34 patients with mild ischemic stroke **Average Age:** 57 **Gender:** 36% female	**Depression and anxiety:** Hamilton Anxiety Rating Scale Hamilton Depression Rating Scale **Activities of daily living, leisure behaviors, and social interaction** (degree to which friends, family or medical staff who provided moral or material support): Electronic interview	Cohort design (data collected baseline and 3 months post hospital discharge) Used an ordinal scale (EMA) 1 day 5x/day and another for 7 days 5x/day @ 3 months later; mood and depression symptoms: 7-point ordinal scale (EMA)
**Fors et al. (2019)**	User evaluation of a novel SMS-based reminder system for supporting post-stroke rehabilitation.	**In Uganda:** ***n*** = 15 people with stroke participated in SMS study. 2 patients dropped out, 2 did not participate in interviews (interview *n* = 11). 11 family members and 4 OT also participated in interviews. **Age/Gender:** No age and gender presented.	**Success rate for performing three daily activities:** Daily SMS responses **Experiences with the SMS tool:** Individual semi-structured interviews to learn user opinions. OT Feedback: questionnaire	Mixed Methods Texts to remind participants to complete daily activities for 8 weeks. Participants responded if completed. Semi-structured interviews w/ participants, family, OTs.
**Lenaert et al. (2020)**	Poststroke Fatigue and Daily Activity Patterns During Outpatient Rehabilitation: An Experience Sampling Method Study	**In Netherlands:** ***n*** = 26 adults with stroke **Average Age:** 55.3 (7.6) **Gender:** Males 13 (50%)	**Fatigue:** Fatigue Severity Scale (FSS). **Anxiety and depression:** Hospital Depression Anxiety Scale (HADS)	Longitudinal observational study. 10 random moments daily for 6 days to measure fatigue and activity

ePub was available in 2019. Light grey shading = included participants with TBI; Dark grey shading = included participants TBI and participants with stroke; No shading (white) = included participants with Stroke.

**Table 2:** Mobile Health Study Results.

Authors [Reference]	Technology Used	EMA	Purpose	Outcome Measures and Results
**Traumatic Brain Injury**				
**Pavliscsk et al.** [33]	**“mCare”** application: bidirectional mobile health system through which participants answered the weekly questionnaires. **2-Way App** Participants completed questionnaires, and participants and providers could exchange messages through app. App developed for community use only	**Yes**	To examine the relationship between health status (behavioral health problems, TBI, and PTSD) and engagement with the mCare mobile app among wounded Service Members rehabilitating in the community.	**Participant engagement:** encompassed exposure to mCare, percentage of questionnaires responded to, and response time. **Acceptable Compliance** In general, participants responded to **≥60% of the questionnaires each week, and in ≤10 h**. Those with behavioral problems, though, had several weeks with a <50% response rate, and had the longest response times. Exposure to mCare, total questionnaires responded to, and response time **did not differ significantly by health status.** **Older age** and **higher General Well-Being Schedule scores** (self-report questionnaire, measuring an individual’s sense of well-being) **were associated with greater and faster responses.** **Psychometric Properties Reported for EMA:** No
**Juengst et al.**	**iPerform:** a user-centered, cross-platform smartphone app. **2-Way App** User interface interacts with participant. Participants sent notifications to complete assessments. Maintains connection with clinician/ researcher portal; could set assessment schedules and sent messages through app. App developed for community use only	**Yes**	To assess the feasibility and validity of an mHealth system for tracking mood-related symptoms after TBI.	Good compliance: Participants correctly completed 73.4% of all scheduled assessments. On average, daily assessments took <2 min to complete. **High satisfaction with smartphone apps:** 6.3 of 7. **Participants found apps easy to use:** 6.2 of 7. **Psychometric Properties Reported for EMA: Yes** **Validity of conducting these assessments via** **smartphone application in this population:** PHQ-9 and GAD-7 scores from phone interview and mobile app were highly correlated—(*r* = 0.81–0.97)
**Wiebe et al.**	iPod Touch & accelerometer **(Custom app loaded on iPod)** **1-Way App:** Participants answered questions via app. App training during initial clinical visit, all measures collected in the community.	**Yes**	To determine feasibility of EMA after youth concussion, gather real-time cognitive and physical activity, and compare objective measures with real-time self-reported symptoms.	**Good compliance:** 82% responded to >80% of prompts. **Cognitive exertion and concussion symptoms were positively correlated** on the same day and on the following 2 days. **Physical activity and concussion symptoms were negatively correlated** on the same day and 2 following days. Most participants (68%) **were no longer reporting symptoms** at the end of the two weeks. **Psychometric Properties Reported for EMA:** No
**Worthen-Chaudhari et al.**	Mobile device app **(Gamified symptom reporting app: Battle Royal Power Pack)** **2-Way App:** Patient symptom reporting via gamification and logged activity. Research coordinator could be invited as an “ally” to send comments, emails, etc. App developed for community use only.	**Yes**	To evaluate if reporting symptoms in a social game designed mobile health app complimented medical care for unresolved symptoms of concussion.	**Feasibility and satisfaction:** Phase I **Positive changes in SCAT-3 concussion symptoms, depression, optimism:** Phase II **No significant correlations** between concussion symptom change and optimism change. **Phase I Barriers to Compliance:** 6 of 14 participants had barriers. **Barriers included:** Discontinuation of medical care 3 Internet challenges: 1 Extracurriculars: 1 Other illness during study: 1 **Phase II Barriers to Compliance:** Not reported Mobile apps that integrate social game mechanics with a heroic narrative may support health management in teenagers with unresolved concussion symptoms. **Psychometric Properties Reported for EMA:** No
**Sufrinko et al.**	Mobile ecological momentary assessment **(mEMA) application**. **1-Way App:** Participants sent notifications to complete assessments. App developed for community use only	**Yes**	Evaluate mEMA as an approach to measure SRC symptoms, explore the relationships between clinical outcomes and mEMA, and determine whether mEMA was advantageous for predicting recovery outcomes compared to traditional symptom report.	**Compliance:** 52.4% for prompts and 50.4% per participant. Symptoms were **lower in the morning** compared with afternoon and evening. **Higher mEMA symptoms were reported during vestibular** compared with physical and sedentary activities. mEMA symptoms were positively associated with PCSS, VOMS, and recovery time, but not neurocognitive scores. **mEMA symptom score was a better predictor of recovery** time than PCSS at either clinic visit. **Psychometric Properties Reported for EMA:** No
**Juengst et al.**	**iPerform:** a user-centered, cross-platform smartphone app. **2-Way App** Participants sent notifications to complete assessments; Clinicians could set custom assessment schedules and sent messages through app. App developed for community use only	**Yes**	To identify potential characteristics that differed between individuals having high versus low variability in symptom reporting.	Significant ***within-person* variability** in reporting across all measures. Women, people with more symptoms, or people with higher cognitive performance report high variability **Compliance** reported in previous publication (Juengst, et al., 2015) **Psychometric Properties Reported for EMA:** No (previously reported in Juengst et al., 2015)
**Lenaert et al.**	Smartphone application **(PsyMate)** **1-Way App:** Participants were prompted with beeps to complete surveys. App developed for community use only.	**Yes**	Investigate the feasibility of using experience sampling in individuals with ABI and explore the usability of experience sampling data	**Compliance:** 71% response rate **High feasibility** and no dropouts, with reports of being user-friendly. Participants reported that questions in PsyMate were a good representation of their experiences. Physical activity and fatigue predicted more negative affect on the same day, but not on later days. Provides a good example of instructions and planning Psychometric Properties Reported for EMA: No
**Cooray et al.**	**Medipal:** smartphone (iPhone and Android) app used to answer questionnaire. **1-Way App:** Participants answered questionnaire via app. App training during hospital stay, not used until after discharge to community.	**No**	To investigate whether automatic assessment of the mRS based on a mobile phone questionnaire may serve an alternative to mRS assessments at clinical visits after stroke.	**Compliance:** 48/62 (77%) completed the assessment **Median 3-month clinical visit mRS:** 2. **Mean 3-month clinical visit mRS:** 2.3. Dichotomized mRS outcome separating functionally independent (mRS score 0–2) from dependent (mRS score 3–5) showed **83% agreement** and unweighted kappa of 0.66 (95% confidence interval 0.45–0.87). **Psychometric Properties Reported for electronic measure:** Yes **62.5% agreement between clinical visit and mobile mRS assessment.** Weighted kappa: 0.89 (95% confidence interval 0.82–0.96). Unweighted kappa: 0.53 (95% confidence interval 0.36–0.70).
**Seo et al.**	**Smartphone application** **2-Way App:** Participants answered questionnaire via app. If data out of normal range, they received an automated alarm, as did researcher/clinician. App developed for community use only.	**Yes**	To assess feasibility of tracking vascular risk factors to improve risk management in persons with stroke	**Fair Compliance: 50% labeled as compliant** (defined by authors as completing >47 of 180 days) Adherence, blood pressure, HbA1c, waist circumference, smoking status. Averages number of days tracked (out of 180) was 60.42 ± 50.17; median = 47, range:1–180. **Target achievement for parameters did not differ based on compliance.** A **shift in risk profiles in a favorable direction was** observed in the entire sample. **Psychometric Properties Reported for EMA:** No
**Villain et al.**	Personal Digital Assistant **(PDA) Palm Tungsten E2.** Software and custom-designed measurement tool not described. **1-Way (PDA)** Participants responded to a rating of mood App was started in the clinic with follow-up in community	**Yes**	Determine the association between the intensity of material and social support measured using EMA in the acute post-stroke phase with the occurrence of depressive symptoms and daily life activities 3 months later	**Acceptable compliance:** 77.3% (33/44) completed 3-month follow-up EMA **Patient perceptions of the quality of moral support** during hospitalization was associated with later **decrease in depressive symptoms** (ᵧ = 0.097, t = −2.141, *p* = 0.041) **and with later increase in ADL participation** (ᵧ = 0.367, t = 2.783, p = 0.011). Greater **perceived material support** was associated with later **increases in ADLs** (ᵧ = 0.333, t = 2.710, *p* = 0.0112). **Psychometric Properties Reported for EMA:** No
**Fors et al.**	**SMS text message via phone** (not smart phone). International commercial **SMS API service called** **Twilio**™ (Twilio.com). **2-Way** Participants responded to text messages and local OTs could use web-based management system to see responses and respond via text Text messaging occurred only in community	**Yes**	Test the usability of a SMS based reminder system to remind patients to perform daily rehab activities and to measure rate of performance from users. Preferences of stakeholders also captured from patients, family members, and therapists.	**Compliance:** not reported **Family and patients had positive opinions.** **Technical issues:** Older clients had more technical challenges. No local telecom operator to support the system. Some clients relied on family members to receive and respond to texts. Can only have 150 characters. Cannot see what they sent and received. **Psychometric Properties Reported for EMA:** No
**Lenaert et al.**	Smartphone application **(PsyMate)** **1-Way App:** Participants were prompted with beeps to complete surveys. App developed for community use only.	**Yes**	To investigate post-stroke fatigue in relation to daily activity patterns during long-term post-stroke rehabilitation.	**Acceptable compliance:** 39 out of 60 questionnaires completed per participant **(65% completion rate).** Participants indicated at least **“some fatigue” in 85% of momentary assessments and more severe fatigue in 61% of momentary assessments.** Participants were relaxing, nothing, or resting in 48.9% of assessments, were doing household activities, self-care, or transport in 24.7% of assessments, and doing something else in 22.3% of assessments. **Fatigue was highest when participants were “doing nothing” or “resting”.** Higher effort and lower enjoyment were associated with higher fatigue. More physical activity was associated with more fatigue. Current fatigue was higher when earlier activities required more effort or more physical activity. **Psychometric Properties Reported for EMA:** No

Light grey shading = included participants with TBI; Dark grey shading=included participants TBI and participants with stroke; No shading (white) = included participants with Stroke.

## Supplementary Materials

The following are available online at https://www.mdpi.com/1660-4601/18/4/2173/s1. Table S1: Database Search Strategy Terms.
