# Supplementary material for: Use of mHealth Technology for Patient-Reported Outcomes in Community-Dwelling Adults with Acquired Brain Injuries: A Scoping Review
Source: Int J Environ Res Public Health. 2021 Feb 23;18(4):2173. doi: 10.3390/ijerph18042173 (PMC7926536; doi:10.3390/ijerph18042173)
Supplement: Supplementary file 1 [file ijerph-18-02173-s001.pdf]

Supplementary Table 1. Database Search Strategy Terms

Search term	AND	Search term
brain injuries<br>OR		Ecological Momentary Assessment<br>OR
brain hemorrhage<br>OR		ecologic* momentary assessment*<br>OR
traumatic brain injuries<br>OR		electronic momentary assessment*<br>OR
diffuse brain injuries<br>OR		community based assessment*<br>OR
traumatic brain injury<br>OR		community based outcome assessment*<br>OR
chronic epilepsy<br>OR		community based measures<br>OR
post-traumatic<br>OR	**AND**	community based outcome measures<br>OR
cerebrovascular trauma<br>OR		cell phone<br>OR
craniocerebral trauma<br>OR		smartphone<br>OR
head injuries<br>OR		
closed head injuries<br>OR		
penetrating<br>OR		
skull fractures<br>OR		
stroke		

Databases searched: Ovid MEDLINE(R)<1946 to August 16, 2019 & MEDLINE InProcess & EPub & Embase & PsycINFO.

Keywords were: [brain injuries/brain hemorrhage/traumatic brain injuries/diffuse brain injuries/traumatic brain injury/chronic epilepsy, post-traumatic OR cerebrovascular trauma/craniocerebral trauma/head injuries/closed head injuries/penetrating/skull fractures OR Stroke] AND [Ecological Momentary Assessment OR ecologic* momentary assessment* OR electronic momentary assessment* OR community based assessment* OR community based outcome assessment* OR community based measures OR community based outcome measures OR cell phone OR Smartphone OR Mobile Applications OR mobile health OR mHealth].
